# Interaction between the PI3K/AKT pathway and mitochondrial autophagy in macrophages and the leukocyte count in rats with LPS-induced pulmonary infection

**DOI:** 10.1515/biol-2022-0588

**Published:** 2023-04-15

**Authors:** Chao Wu, Lianghua Guo, Xirennayi Muhataer, Qifeng Li, Zhichuang Lian, Yafang Li, Wenyi Wang, Wei Ding, Yuan Zhou, Xiaohong Yang, Muzhi Chen

**Affiliations:** Department of Respiratory and Critical Care Medicine, People’s Hospital of Xinjiang Uygur Autonomous Region, No. 91 Tianchi Road, Tianshan District, 830001 Urumqi, China; Department of Respiratory Medicine, Mindong Hospital Affiliated to Fujian Medical University, 355000 Fu’an City, China; Xinjiang Institute of Pediatrics, Children’s Hospital of Xinjiang Uygur Autonomous Region, Urumqi 830054, China; Department of Rheumatology, The Second Affiliated Hospital of Zhejiang Chinese Medical University, No. 318, Chaowang Road, Gongshu District, 310005 Hangzhou, China

**Keywords:** severe pneumonia, leukocyte, mitochondrial autophagy, PI3K-AKT-mTOR pathway, molecule

## Abstract

This study examined the effects of the PI3K/AKT pathway and mitochondrial autophagy in macrophages and the leukocyte count after pulmonary infection. Sprague‒Dawley rats were subjected to tracheal injection of lipopolysaccharide (LPS) to establish animal models of pulmonary infection. By inhibiting the PI3K/AKT pathway or inhibiting/inducing mitochondrial autophagy in macrophages, the severity of the pulmonary infection and the leukocyte count were altered. The PI3K/AKT inhibition group did not show a significant difference in leukocyte counts compared with the infection model group. Mitochondrial autophagy induction alleviated the pulmonary inflammatory response. The infection model group had significantly higher levels of LC3B, Beclin1, and p-mTOR than the control group. The AKT2 inhibitor group exhibited significantly increased levels of LC3B and Beclin1 compared with the control group (*P* < 0.05), and the Beclin1 level was significantly higher than that in the infection model group (*P* < 0.05). Compared with the infection model group, the mitochondrial autophagy inhibitor group exhibited significantly decreased levels of p-AKT2 and p-mTOR, whereas the levels of these proteins were significantly increased in the mitochondrial autophagy inducer group (*P* < 0.05). PI3K/AKT inhibition promoted mitochondrial autophagy in macrophages. Mitochondrial autophagy induction activated the downstream gene *mTOR* of the PI3K/AKT pathway, alleviated pulmonary inflammatory reactions, and decreased leukocyte counts.

## 1 Introduction

Currently, infection-induced deaths account for approximately one-fourth of the total mortality worldwide; in recent years, the incident rate of infection has remained high due to complications, the use of glucocorticoids, low immune function, bacterial resistance, and invasive operations [1]. After infection, most patients develop leukocytosis, which is the result of the rapid initiation of the innate immune response; however, some patients exhibit leukocytopenia [2]. In our clinical practice, patients with leukocytopenia after infection are not rare. Moreover, in these patients, infection symptoms are more severe and more difficult to control because pathogens can more easily evade attacks from immune cells, and consequently, treatments tend to be longer [3].

Infections, whether with leukocytosis or leukocytopenia, are primarily caused by pathogens, which mainly include viruses and bacteria. After infection occurs, the innate immune response, also termed natural immunity, is activated first. Innate immunity plays a critical role in acute infection, and neutrophils, lymphocytes, eosinophils, and basophils are important innate immune cells. These cells arrive at the infection site through rolling, adhesion, tight binding, cell overflow, and migration and exert direct immune effects by engulfing, killing, and digesting the invaders through the release of reactive oxygen substances and antimicrobial cleaved granule proteins such as peroxidase, lysozyme, alkaline phosphatase, and acid hydrolase [4,5]. In patients with leukocytopenia after infection [6], however, cell migration disorder, abnormal bone marrow proliferation, decreased chemokine secretion, and the presence of abnormal receptors on the surface of immune cells may occur [7].

According to our previous work [3], abnormal chemokine secretion, cell migration disorder, ubiquitination modification disorder, and decreased oxidative stress may participate in the development of postinfection leukocytopenia; compared with the control group, the leukocytopenia group showed hypermethylation of the thymoma viral proto-oncogene 2 (*AKT2*) gene, whereas leukocytosis induced hypomethylation. AKT2 serves as a key protein in the PI3K/AKT pathway [8]. After pathogens invade the body, innate immune cells, such as dendritic cells, macrophages, NK cells and neutrophils, are activated first [9]. Although immune cells do not express specific antigen recognition receptors, they recognize immunologically activated receptors via pattern recognition and then secrete corresponding cytokines, thereby exerting immediate immune effects. However, the target cells of the PI3K/AKT pathway, as well as the mechanism underlying the interaction between these cells, remain to be examined [10,11].

PI3K/AKT is a typical autophagy signaling pathway [12]. Autophagy has a direct effect on microbial infection and can selectively remove intracellular microorganisms; autophagy even functions as an independent natural defense barrier [13]. The PI3K/AKT pathway is associated with mitochondrial autophagy in macrophages [14], and the mutual recruitment and accumulation of macrophages and neutrophils at the site of inflammation promote cooperation between phagocytes. At the infection site, a large amount of chemokines are released, which attract leukocytes to accumulate; the recruited immune cells further release chemokines to promote the generation of leukocytes, which are then released into blood, thereby forming a complex chemokine network system [15].

Because the PI3K/AKT signaling pathway might participate in the development of postinfection leukocytopenia and the PI3K/AKT pathway is closely associated with mitochondrial autophagy in macrophages, we hypothesized that the PI3K/AKT pathway participates in the development of leukocytopenia by inducing mitochondrial autophagy. To verify this hypothesis, we conducted the current study, which aimed to examine the molecular mechanism of leukocytopenia in a rat model of lipopolysaccharide (LPS)-induced pulmonary infection by interfering in the PI3K/AKT pathway and macrophage autophagy.

## 2 Materials and methods

### 2.1 Animals

Specific pathogen-free (SPF) Sprague‒Dawley rats were purchased from the Teaching and Scientific Research Condition Guarantee Center of Xinjiang Medical University (license no., SCXK(Xin)2018-0002). The age of the animals ranged from 6 weeks to 8 weeks, with body masses of 180–220 g. The rats were fed at the Animal Center of Xinjiang Medical University. The feeding environment was as follows: temperature, 21–25°C; relative humidity, 50–70%; ventilation frequently, 1–2 times per day; air velocity, 0.1–0.2 cm/s; environmental noise, under 60 dB; and illumination, 15–20 lx. The rats were used for the experiment after 1 w of quarantine.


**Ethical approval:** The research related to animal use has been complied with all the relevant national regulations and institutional policies for the care and use of animals. The procedures of this study were approved by the Ethics Committee of People’s Hospital of Xinjiang Uygur Autonomous Region (approval no., 2020056). This study is reported in accordance with ARRIVE guidelines.

### 2.2 Groupings

The animals were randomized into five different groups, with six animals of the same sex in each group. To avoid artificial influences on grouping outcomes, a random number table was used. The detailed treatments for each group are described below.

#### 2.2.1 The negative control group

The animals were subjected to tracheal injection of 5 mg/kg saline and intraperitoneal injection of 25 mg/kg saline. Saline (10 mg/kg day) was administered by gavage. After 6 days of treatment, experiments were then performed.

#### 2.2.2 The infection model group

The animals were subjected to tracheal injection of 5 mg/kg LPS (Sigma; article no., L2880) and intraperitoneal injection of 25 mg/kg saline. Saline (10 mg/kg day) was administered by gavage. After 6 days of treatment, experiments were then performed.

#### 2.2.3 AKT2 inhibitor (MCE; article no., HY-13260) group

The animals were subjected to tracheal injection of 5 mg/kg LPS (Sigma; article no., L2880) and intraperitoneal injection of the AKT2 inhibitor (25 mg/kg). After 6 days of treatment, experiments were performed.

#### 2.2.4 The mitochondrial autophagy inhibitor group

The animals were subjected to tracheal injection of 5 mg/kg LPS followed by gavage administration of 10 mg/kg day cyclosporin A (CsA; MCE; article no., HY-B0579). After 6 days of treatment, experiments were then performed.

#### 2.2.5 The mitochondrial autophagy inducer group

The animals were subjected to tracheal injection of 5 mg/kg LPS followed by intraperitoneal injection of 4 mg/kg day carbonyl cyanide 3-chlorophenylhydrazone (CCCP; MCE; article no., HY-100941). After 6 days of treatment, experiments were then performed.

### 2.3 Whole blood leukocyte counting

The rats were anesthetized intraperitoneally with 10% chloral hydrate (0.3 mL/100 g). The abdominal skin was cut open in a “V” shape. A blood-collecting needle was inserted into the branch of the common iliac artery and then pushed centripetally along the abdominal aorta. A total of 0.5 mL of whole blood was collected from each group. Hemolysin (1×) (3 mL) was added. After vortex mixing, the sample was placed in the dark for 10 min. After sufficient lysis, the sample was centrifuged at 1,500 rpm for 5 min. The supernatant was removed, and the residue was washed twice with PBS. Leukocytes were isolated. PBS (0.5 mL) was added to resuspend the cells. A cover glass was placed in the center of the cell counting plate. An appropriate volume of the cell suspension was applied. The total number of cells in the five central squares was counted. The number of leukocytes was calculated based on the following formula:

\text{Number}\hspace{.25em}\text{of}\hspace{.25em}\text{leukocytes}/\hspace{-.35em}\text{L}\hspace{.25em}=N/5\times 25\times 10\times {10}^{6},]

where *N* is the total cell number within the five squares.

### 2.4 Flow cytometry

Macrophages were isolated using flow cytometry. A total of 4 mL of whole blood was collected from each group, which was added to four flow tubes (1 mL each). Then, 3 mL of 1× hemolysin was added, and the solution was well mixed. The solution was placed in the dark for 10 min. After complete lysis, centrifugation at 1,500 rpm for 5 min was performed. The supernatant was discarded, and PBS washes were performed twice. Leukocytes were separated. PBS (1 mL) was added to resuspend the cells. Afterward, 15 μl of CD14 antibody was added. The solution was incubated at 4°C in the dark for 15 min. After centrifugation at 1,500 rpm for 5 min, the supernatant was removed. Prechilled serum-free RPMI 1640 culture medium (5 mL) was used to resuspend the cells. CD14 single-positive cells were separated using flow cytometry. The isolated cells were subjected to statistical analysis for subsequent gene and protein detection.

### 2.5 Hematoxylin–eosin (H&E) staining

The animals were anesthetized with 10% chloral hydrate (0.3 mL/100 g) and then killed by decapitation. Pulmonary tissue was isolated for paraffin embedding. After being dehydrated, the sections were stained with a hematoxylin aqueous solution for several minutes. After color separation with acidic water and ammonia in water, the specimen was dehydrated with 70 and 90% ethanol for 10 min each. Afterward, eosin staining was performed for 2–3 min. The sections were dehydrated with pure ethanol, made transparent with xylene, and then sealed with Canadian gum.

The stained sections were scored based on neutrophil infiltration, airway epithelial cell injury, interstitial edema, pulmonary hyaline membrane, and bleeding, and the scoring criteria were as follows [16]: normal, 0 points; minor changes, 1 point; mild changes, 2 points; moderate changes, 3 points; and severe changes, 4 points.

### 2.6 qRT‒PCR

Total RNA was extracted using the TRIzol method, and gene primers were designed with Primer 5. The primer sequences (5′- to -3′) are listed as follows:

AKT2, GGAGCTCTGTTAGCACCGTT (F) and AGTGGAAATCCAGTTCCGAGC (R) (product size, 101 bp);

LC3B, GAGCGAGAGAGATGAAGACGG (F) and ACGTCCCTTTTTGCCTTGGT (R) (product size, 133 bp);

PI3K, ACATCGACCTACACTTGGGG (F) and TCCCCTCTCCCCAGTAGTTT (R) (product size, 140 bp);

mTOR, AGAACCTGGCTCAAGTACGC (F) and AGGATGGTCAAGTTGCCGAG (R) (product size, 114 bp); and

GAPDH, CAGGGCTGCCTTCTCTTGTG (F) and GATGGTGATGGGTTTCCCGT (R) (product size, 172 bp).

A total of 20 µL of the reference reaction system for cDNA synthesis included 7 µL of total RNA, 1 µL of random primers (0.1 g/l), 10 µL of 2× TS reaction mix, 1 µL of TransScript@RT/RI enzyme mix, 1 µL of gDNA Remover, and 20 µL of RNase-free water. The PCR system was prepared, and a total of 10 µL of the system included 1 µL of cDNA, 5 µL of 2× SYBR Green Select Mix, 0.7 µL of forward primer, 0.7 µL of reverse primer, 0.05 µL of ROX, and RNase-free water. The reaction conditions consisted of 95°C for 5 min followed by 40 cycles of 95°C for 5 sec and 60°C for 30 s. The CT values were determined, β-actin was used as the internal reference, and the relative expression of *Orexin-A* was calculated using the 2^−△△Ct^ method.

### 2.7 Western blot analysis

Cells were washed three times with prechilled PBS and then lysed for protein extraction. The protein concentration was measured with the bicinchoninic acid (BCA) method. Afterward, electrophoresis (the formula for preparing separating gel and concentrated gel was summarized in Supplementary File 1), and membrane transfer were performed. Nonfat milk (50 g/L) was applied for 2 h of blocking, and then, the membrane was washed three times with TBST (10 min each time). Primary and secondary antibodies were applied according to the instructions. The detailed information of the antibodies was as follows: mouse beta-actin antibody (dilution, 1:15,000; 100166-MM10; Sinobiological, Beijing, China), recombinant anti-AKT2 (1:5,000; ab131168; Abcam), anti-AKT2 (phospho S474) (1:5,000; ab38513; Abcam), PI3 kinase p110α (C73F8) rabbit mAb (1:5,000; 4249S; CST), mTOR (7C10) rabbit mAb (1:5,000; 2983S; CST), phospho-mTOR (Ser2448) antibody (1:5,000; 2971S; CST), recombinant anti-LC3B (1:5,000; ab192890; Abcam), and recombinant anti-Beclin 1 (1:5,000; ab210498; Abcam). Chemiluminescence reactions were performed. The gray values of the developed protein bands were detected with ImageJ, and statistical analyses were performed based on the gray value of the internal reference.

### 2.8 Statistical analysis

All data are presented as the mean ± standard deviation (\bar{x}\pm s)] , and analyses were performed with SPSS 19.0. The student *t*-test was used to compare two groups, and the Student–Newman–Keuls test was used for comparisons among three or more groups. *P* < 0.05 (two-tailed) was considered significantly different. Graphs were plotted with GraphPad Prism 5.0.

## 3 Results

### 3.1 H&E staining

**Figure 1:**
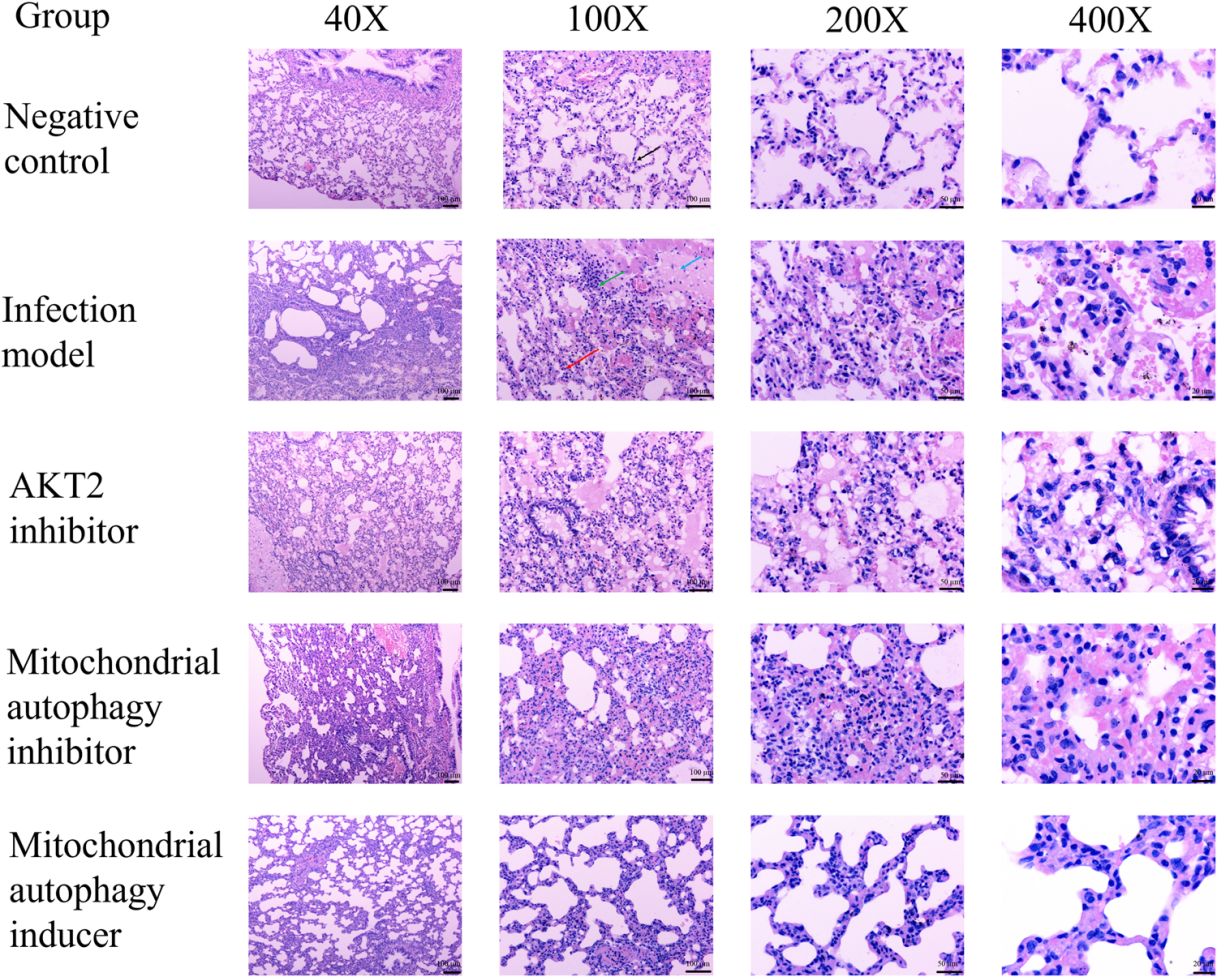
Hematoxylin–eosin staining outcomes of the pulmonary tissue in the different groups (*n* = 3). The black arrow indicates normal alveolus, the green arrow indicates inflammatory cell infiltration, the blue arrow indicates inflammatory exudation, and the red arrow indicates alveolar septal edema.

**Figure 2:**
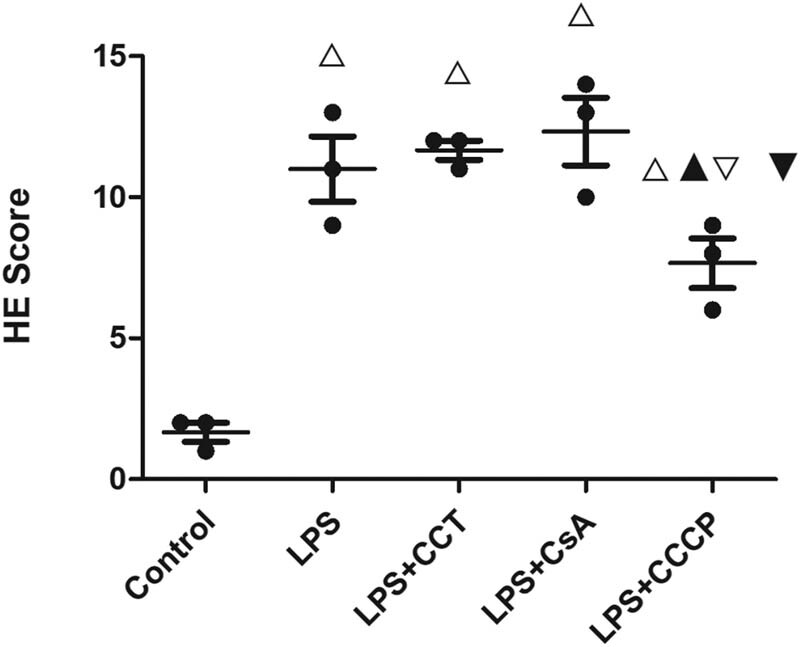
Hematoxylin–eosin staining scores of the pulmonary tissue in different groups (*n* = 3). One-way analysis of variance is used for comparison among groups. △*P* < 0.05, vs the control group; ▲*P* < 0.05, vs the infection model group; ▽*P* < 0.05, vs the AKT2 inhibitor group; and ▼*P* < 0.05, vs the mitochondrial autophagy inhibitor group.

In the control group, pulmonary tissue structure was normal, and there was a small number of chronic inflammatory cells in the stroma (Figure 1). In the model group, part of the bronchus exhibited membranous epithelial erosion and loss, and the infiltration with a large amount of chronic inflammatory cells was observed in the submucosa. The alveolar septa were thickened, and edema and the infiltration of many neutrophils were observed. A large number of red blood cells were observed in the stroma and alveolar cavity, and bleeding foci formed in some areas. The walls of some alveoli were dilated and fractured. Compared with the model group, the AKT2 inhibitor group and the mitochondrial autophagy inhibitor group did not show noticeable pathological changes. In the mitochondrial autophagy inducer group, the severity of pulmonary tissue injury was lower than that in the model group. The H&E scores are shown in Figure 2.

### 3.2 Leukocyte counts

The infection model group had a significantly higher leukocyte count than the control group (10.575 ± 2.447 vs 6.800 ± 1.047; *P* < 0.05) (Table 1). The cell counts in the AKT2 inhibitor group and mitochondrial autophagy inhibitor group were 10.363 ± 2.005 and 11.575 ± 1.946, respectively, both of which showed significant differences compared with the control group (*P* < 0.05). No significant differences were observed among the infection model group, the AKT2 inhibitor group, and the mitochondrial autophagy inhibitor group. The cell count in the mitochondrial autophagy inducer group was 4.563 ± 1.298, which was significantly lower than that in any of the other groups (*P* < 0.05).

**Table 1:** Whole blood leukocyte counts in the different groups ( \overline{x}\pm s] , *n* = 6)

Group	Leukocyte count (10^9^/L)
Negative control	6.800 ± 1.047
Infection	10.575 ± 2.447^△^
AKT2 inhibitor	10.363 ± 2.005^△^
Mitochondrial autophagy inhibitor	11.575 ± 1.946^△^
Mitochondrial autophagy inducer	4.563 ± 1.298^△▲▽▼^

△*P* < 0.05, vs the control group; ▲*P* < 0.05, vs the infection model group; ▽*P* < 0.05, vs the AKT2 inhibitor group; and ▼*P* < 0.05, vs the mitochondrial autophagy inhibitor group.

### 3.3 Gene expression in CD14+ monocyte/macrophages

The RNA expression of the investigated genes in blood monocyte/macrophages was analyzed (Table 2). *AKT2* expression in the AKT2 inhibitor group was significantly lower than that in any of the other groups (*P* < 0.05). The control group, the infection model group, and the AKT2 inhibitor groups did not show significant differences in *LC3B* expression (*P* > 0.05). Compared with these groups, the mitochondrial inhibitor group exhibited significantly lower *LC3B* expression, whereas the mitochondrial inducer group showed significantly higher expression (*P* < 0.05). There were no significant differences in *PI3K* expression among the five groups (*P* > 0.05). mTOR expression in the control group and the infection model group was 1.069 ± 0.470 and 1.400 ± 0.544, respectively, and no significant difference was observed between the groups (*P* > 0.05). *mTOR* expression in the AKT2 inhibitor group and the mitochondrial autophagy inhibitor group was 0.761 ± 0.213 and 0.803 ± 0.286, respectively, and showed significant differences compared with the infection model group (*P* < 0.05). *mTOR* expression in the mitochondrial autophagy inducer group was 1.360 ± 0.479, which was comparable to that in the control group and infection model group (*P* > 0.05) but was significantly higher than that in the AKT2 inhibitor group and mitochondrial autophagy inhibitor group (*P* < 0.05).

**Table 2:** Gene expression in CD14+ monocyte/macrophages in the different groups ( \overline{x}\pm s] , *n* = 6)

Group	*AKT2*	*LC3B*	*PI3K*	*mTOR*
Negative control	1.082 ± 0.458	1.074 ± 0.437	1.040 ± 0.307	1.069 ± 0.470
Infection	1.249 ± 0.252	1.257 ± 0.358	1.258 ± 0.254	1.400 ± 0.544
AKT2 inhibitor	0.849 ± 0.239^▲^	1.280 ± 0.301	1.010 ± 0.284	0.761 ± 0.213^▲^
Mitochondrial autophagy inhibitor	1.164 ± 0.392	0.715 ± 0.262^△▲▽^	1.041 ± 0.382	0.803 ± 0.286^▲^
Mitochondrial autophagy inducer	1.169 ± 0.252	1.466 ± 0.363^△▲▽▼^	1.090 ± 0.235	1.360 ± 0.479^▽▼^

△*P* < 0.05, vs the control group; ▲*P* < 0.05, vs the infection model group; ▽*P* < 0.05, vs the AKT2 inhibitor group; and ▼*P* < 0.05, vs the mitochondrial autophagy inhibitor group.

### 3.4 Protein levels in CD14+ monocyte/macrophages

**Figure 3:**
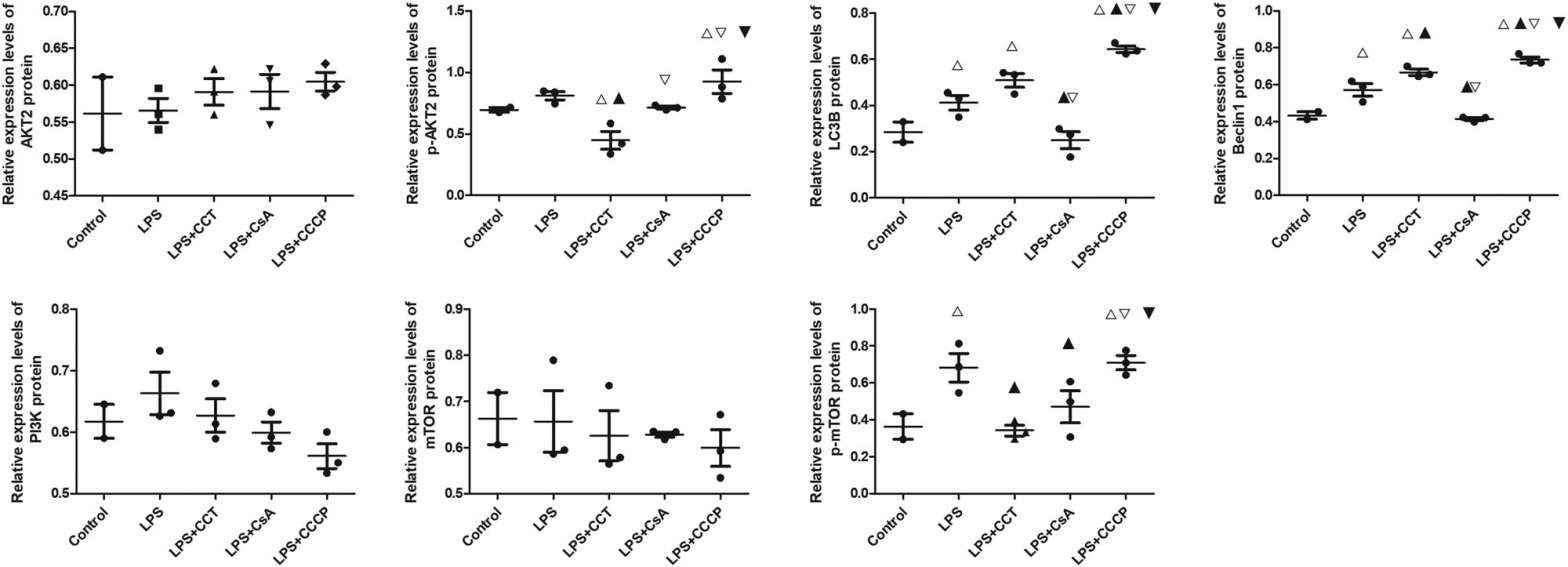
Protein levels in peripheral CD14+ monocyte/macrophages in the different rat groups. Each group contains three animals, and flow cytometry is used for western blot analysis of CD14+ CELLS, with three repetitions in each group. One-way analysis of variance is used for comparison among groups. A, Splicing band diagram of the target proteins. B, The levels of different proteins in macrophages. B1-5, The levels of the key proteins in the PI3K/AKT pathway. B6-7, Markers of mitochondrial autophagy in macrophages. △*P* < 0.05, vs the control group; ▲*P* < 0.05, vs the infection model group; ▽*P* < 0.05, vs the AKT2 inhibitor group; and ▼*P* < 0.05, vs the mitochondrial autophagy inhibitor group.

Protein levels in blood monocyte/macrophages were analyzed (Figure 3; uncropped gels and blots are shown in Supplementary File 2). No significant differences in the levels of AKT2, PI3K, or mTOR were observed among the five groups. Compared with the control group, the AKT2 inhibitor group showed significantly higher levels of LC3B and Beclin1 (*P* < 0.05). The Beclin1 level was significantly higher than that in the infection model group (*P* < 0.05). Compared with the infection model group, the mitochondrial autophagy inhibitor group exhibited significantly lower levels of p-AKT2 and p-mTOR, whereas the mitochondrial autophagy inducer group exhibited significantly higher levels of these proteins (*P* < 0.05).

## 4 Discussion

To date, the mechanism underlying the development of leukocytopenia after the infection has not been completely clarified. AKT participates in the innate immune response and inflammatory reactions [17]; the PI3K/AKT signaling pathway is a tyrosine kinase cascade and signal transduction pathway [18]. PIP3 serves as an important second messenger in cellular signaling pathways. PIP3 recruits signaling molecules containing PH domains (e.g., AKT) onto the plasma membrane for activation, which in turn initiates the downstream signaling pathway. AKT is a direct target gene of PI3K. After AKT is recruited by PI3K-medicated second messengers to the cell membrane, its conformation is changed [19]. Activated AKT inhibits the inflammatory reaction in mice and rabbits with LPS-induced sepsis, and the possible mechanisms may be attributed to increased IL-12, TNF-α, and IL-6 (proinflammatory cytokines) levels and decreased IL-10 (an anti-inflammatory cytokine) levels after PI3K or AKT is inhibited [19,20]. Therefore, it is reasonable to expect that postinfection leukocytopenia is associated with the inhibition of the PI3K/AKT pathway. However, our study showed that the leukocyte count was significantly increased in rats after LPS-induced pulmonary infection, but AKT2 inhibition did not result in leukocytopenia, which suggested that AKT2 and its downstream cytokines were not the key genes that led to leukocytopenia after infection and that postinfection leukocytopenia might be the consequence of decreased AKT2 activity due to immunosuppression. We found that compared with the control group, the infection model group, which was induced by LPS, showed significantly increased p-mTOR levels in macrophages, as well as increased levels of LC3B II and Beclin1 (mitochondrial autophagy markers in macrophages); after AKT2 inhibition, LC3B II and Beclin1 levels were further increased, although the pathological changes were not noticeable, compared with those in the infection model group, suggesting that AKT2 inhibition might further exacerbate postinfection mitochondrial autophagy. mTOR participates in the inflammatory reaction and serves as a key protein in the PI3K/AKT downstream signaling pathway; it also serves as an important negative regulator of autophagy [18]. Therefore, we hypothesized that postinfection leukocytopenia was associated with autophagy mediated by the PI3K-AKT-mTOR signaling pathway.

The PI3K/AKT signaling pathway is the only known autophagy-inhibiting signal transduction pathway. This pathway regulates the levels of multiple inflammatory factors in sepsis; it may be of great importance in regulating autophagy [21]. mTOR is a conserved serine-threonine protein kinase and the substrate of AKT after activation. Activated AKT can phosphorylate mTOR to increase its activity. In this study, AKT2 inhibition greatly strengthened mitochondrial autophagy in macrophages after pulmonary infection in rats. According to the literature [14], activation of the PI3K/AKT pathway promotes the levels of the key proteins associated with granulocyte autophagy in macrophages, such as Parkin and PINK1; after the pathway is inhibited, however, this beneficial effect disappears. Our results were consistent with the reported results. Currently, it is thought that mTOR regulates autophagy in two ways [22]. First, mTOR directly acts on ATG proteins. mTOR can phosphorylate multiple autophagy proteins to block the dimerization of ULK1 and impair the formation of autophagosomes during the induction period, thereby inhibiting autophagy. Second, mTOR is at the intersection of multiple signaling pathways and can integrate nutrient and growth factor signals; mTOR promotes transcription and translation to adjust the life cycle of cells [22]. Based on these theories, we performed further intervention in rats by inhibiting/inducing mitochondrial autophagy and observed the effects of the changes in mitochondrial autophagy in macrophages on the PI3K/AKT pathway and leukocyte counts after LPS-induced pulmonary infection. We found that autophagy inhibition did not noticeably alter pulmonary inflammation, whereas autophagy induction alleviated the severity of inflammation to some extent. In addition, autophagy inhibition did not significantly increase leukocyte counts, whereas autophagy induction significantly decreased leukocyte counts. Our findings were consistent with the reported theory: after bacteria invade the body, an immune response is initiated, and neutrophils immediately penetrate the vascular wall to arrive at the infection site [23]. To remove the bacteria, the demand for granulocytes at the infection site increases, and autophagy in increased in the immune cells at the site, which increases the consumption rate of neutrophils; under these conditions, if immune cells in blood vessels cannot be effectively supplemented, leukocytopenia will occur [23,24].

Organisms are constantly altered, and cells and cellular components are constantly being remolded and recycled. Therefore, low levels of basic autophagy occur to maintain homeostasis. However, autophagy can be highly induced, particularly under intracellular and/or extracellular stress, such as nutrient deficiency, hypoxia, oxidative stress, metabolic imbalance, oncogene activation, and pathogen infection; these conditions initiate autophagy to protect the organism [25]. Macrophages are usually present in the lung stroma and alveoli, and these cells are important for initiating lung inflammation during injury, such as infection. When inflammation occurs, more macrophages gather in the lungs to maintain and exacerbate inflammatory damage. In LPS-induced inflammation, macrophage autophagy inhibits inflammatory reactions; in contrast, in mice with autophagy deficits, a large number of abnormal mitochondria accumulate in macrophages, which further activates the NALP3 inflammasome and mitochondrial reactive oxygen species (ROS) to induce inflammation, increasing the mortality of the mice [26,27]. After macrophages were treated with Toll-like receptor ligands, proinflammatory factors could bind to autophagosomes, while rapamycin (RAPA) could degrade these factors and inhibit inflammatory factor secretion [28]. *Beclin1* (an autophagy gene) knockout could increase the probability of septicemia in mouse models of cecal ligation and puncture (CLP), whereas CO could increase the expression of *Beclin1* in mice and in macrophages, thereby promoting macrophage phagocytosis of bacteria [29,30]. In this study, we found that autophagy inhibition decreased the expression of p-mTOR in macrophages, whereas autophagy induction increased the expression of p-AKT2 and p-mTOR. As a typical autophagy inhibition signaling pathway, PI3K/AKT plays a role throughout the development of pulmonary infection. Basic research has shown that dysregulated autophagy activation in the early stage will lead to autophagy disorder and immunosuppression with disease progression, which in turn affects the function of multiple organs [26]. In our study, after pulmonary infection, mitochondrial autophagy induction decreased leukocyte counts, and pathological examination showed the alleviation of pulmonary inflammation. Therefore, targeting different elements in the autophagy pathway and altering the activity of different sections of the pathway during the course of the disease may be a strategy in the treatment of sepsis. At present, mTOR serves as a target that is easily regulated according to research in the field of cancer and other fields; by inhibiting mTOR, autophagy can be effectively activated; however, whether this technique is applicable to preventing severe infection from progressing to sepsis and multiple organ dysfunction remains to be examined in future basic research and clinical trials [31,32].

In our previous study, after bacterial infection, patients with leukocytopenia exhibited abnormal methylation of *AKT2* [3]. This study aimed to examine the molecular mechanism underlying postinfection leukocytopenia, focusing on the innate immune system after infection, and one of the hypotheses was that the PI3K/AKT pathway regulates macrophage autophagy via the key protein AKT2 and leads to postinfection leukocytopenia. However, our results suggested that the PI3K/AKT pathway was not the cause of this condition and that leukocytopenia and abnormal *AKT2* hypermethylation were possibly manifestations of immunosuppression during bacterial infection. Although we did not obtain positive results to verify this hypothesis, our results could partially explain the regulatory mechanism underlying the interaction between the PI3K/AKT pathway and mitochondrial autophagy after pulmonary infection.

Therefore, this study suffered from limitations, particularly regarding mitochondrial autophagy inhibition/induction. We altered mitochondrial autophagy in macrophages, and mitochondrial autophagy in other immune cells was altered accordingly, particularly in neutrophils, whose immune response is a crucial immune link after pulmonary bacterial infection. According to the literature, the effect of altering neutrophil autophagy on inflammation was opposite to that of macrophages [33,34]. In LPS-induced inflammatory reactions, macrophage autophagy could inhibit inflammatory reactions, whereas in mice with autophagy deficits, abnormal mitochondrial accumulation was observed in macrophages, which further activated NALP3 inflammasomes and ROS to induce inflammation, increasing the mortality of the mice [33]. In mouse models of LPS-induced ALI, neutrophil autophagy is required for the activation of cells and the release of particulate contents from these cells; during ALI, the increased autophagy in neutrophils increases the release of particulate contents, and therefore, inhibiting neutrophil autophagy and Atg5 (an autophagy gene) ameliorate the effect of LPS and fMLP on the release of MPO from neutrophils and inhibit the release of particulate contents, thereby alleviating ALI [34]. In addition, our study showed that patients with leukocytopenia after infection exhibited abnormal hypermethylation of *AKT2* [3] and that inhibiting the PI3K/AKT pathway could strengthen mitochondrial autophagy in macrophages, which in turn alleviated the pulmonary inflammatory response. Encouraged by these results, it would be reasonable to hypothesize that the probability of critical illness, such as sepsis, might be decreased in patients with leukocytopenia due to immunosuppression. This concept is worth further exploration in the future. In addition, the results obtained in this study were based on animal experiments, and the sample size was small. Therefore, the results of this study could only reflect a trend, which needs to be validated by increasing the sample size or conducting clinical trials.

PI3K/AKT inhibition promotes mitochondrial autophagy in macrophages. Mitochondrial autophagy induction alleviates the pulmonary inflammatory response and decreases leukocyte counts. Furthermore, mitochondrial autophagy induction strengthens the activity of mTOR, a downstream gene of the PI3K/AKT pathway, which may be the result of the negative regulation of this pathway. The results of this study provide a theoretical foundation for preventing the progression of leukocytopenia after pulmonary infection to severe conditions such as sepsis and multiple organ dysfunction. However, whether the ideal therapeutic target is mTOR remains to be experimentally validated.

## Supplementary Material

Supplementary material
