# Supplementary material for: Interaction between the PI3K/AKT pathway and mitochondrial autophagy in macrophages and the leukocyte count in rats with LPS-induced pulmonary infection
Source: Open Life Sci. 2023 Apr 15;18(1):20220588. doi: 10.1515/biol-2022-0588 (PMC10106970; doi:10.1515/biol-2022-0588)
Supplement: Supplementary material [file biol-2022-0588-sm.pdf]

Supplementary material

Table S1: Formula for preparing separating gel and concentrated gel

5% Separating gel		5% Concentrated gel	
**Reagent**	**Volume**	**Reagent**	**Volume**
Deionized water (mL)		Deionized water (mL)	4.0
30% acrylamide (mL)		0% acrylamide (mL)	1.0
1.5 mol/l Tris-Cl (pH 8.8) (mL)		1.0 mol/l Tris-Cl (pH 6.8) (mL)	1.0
10% SDS (mL)		10% SDS (μL)	80.0
10% AP (mL)		10% AP (μL)	60.0
TEMED (μL)		TEMED (μL)	8.0
Total volume (mL)	12.0	Total volume (mL)	6.0
